# SERPINB3 in the Chicken Model of Ovarian Cancer: A Prognostic Factor for Platinum Resistance and Survival in Patients with Epithelial Ovarian Cancer

**DOI:** 10.1371/journal.pone.0049869

**Published:** 2012-11-21

**Authors:** Whasun Lim, Hee Seung Kim, Wooyoung Jeong, Suzie E. Ahn, Jinyoung Kim, Yong Beom Kim, Min A. Kim, Mi-Kyung Kim, Hyun Hoon Chung, Yong Sang Song, Fuller W. Bazer, Jae Yong Han, Gwonhwa Song

**Affiliations:** 1 WCU Biomodulation Major, Department of Agricultural Biotechnology, Seoul National University, Gwanak-gu, Seoul, Korea; 2 Department of Obstetrics and Gynecology, Seoul National University College of Medicine, Jongno-Gu, Seoul, Korea; 3 Department of Obstetrics and Gynecology, Seoul National University Bundang Hospital, Bundang-Gu, Seoungnam, Korea; 4 Department of Pathology, Seoul National University College of Medicine, Jongno-Gu, Seoul, Korea; 5 Cancer Research Institute, Seoul National University College of Medicine, Seoul, Korea; 6 Center for Animal Biotechnology and Genomics and Department of Animal Science, Texas A&M University, College Station, Texas, United States of America; Beckman Research Institute of City of Hope, United States of America

## Abstract

Serine protease inhibitors (SERPINs) appear to be ubiquitously expressed in a variety of species and play important roles in pivotal physiological processes such as angiogenesis, immune responses, blood coagulation and fibronolysis. Of these, squamous cell carcinoma antigen 1 (SCCA1), also known as a SERPINB3, was first identified in squamous cell carcinoma tissue from the cervix of women. However, there is little known about the SERPINB3 expression in human epithelial ovarian cancer (EOC). Therefore, in the present study, we investigated the functional role of *SERPINB3* gene in human EOC using chickens, the most relevant animal model. In 136 chickens, EOC was found in 10 (7.4%). *SERPINB3* mRNA was induced in cancerous, but not normal ovaries of chickens (P<0.01), and it was abundant only in the glandular epithelium of cancerous ovaries of chickens. Further, several microRNAs, specifically *miR-101, miR-1668* and *miR-1681* were discovered to influence *SERPINB3* expression via its 3′-UTR which suggests that post-transcriptional regulation influences *SERPINB3* expression in chickens. SERPINB3 protein was localized predominantly to the glandular epithelium in cancerous ovaries of chickens, and it was abundant in the nucleus of both chicken and human ovarian cancer cell lines. In 109 human patients with EOC, 15 (13.8%), 66 (60.6%) and 28 (25.7%) patients showed weak, moderate and strong expression of SERPINB3 protein, respectively. Strong expression of SERPINB3 protein was a prognostic factor for platinum resistance (adjusted OR; odds ratio, 5.94; 95% Confidence Limits, 1.21–29.15), and for poor progression-free survival (PFS; adjusted HR; hazard ratio, 2.07; 95% CI; confidence interval, 1.03–4.41). Therefore, SERPINB3 may play an important role in ovarian carcinogenesis and be a novel biomarker for predicting platinum resistance and a poor prognosis for survival in patients with EOC.

## Introduction

Epithelial ovarian cancer (EOC) is the 7^th^ leading cause of cancer-related deaths in women worldwide [1], [2]. The clinical importance has grown to a greater extent because there are no relevant symptoms and no effective screening methods for early detection [3], which leads to International Federation of Gynecologist (FIGO) Stages III-IV disease at the time of diagnosis in over 75% of patients with EOC [4]. Although early-stage disease, well differentiation, platinum sensitivity and optimal cytoreductive surgery have been suggested as favorable prognostic factors, but their value in prognosis has not been improved markedly [5]. Therefore, the early detection of EOC and prediction of prognosis for patient survival using specific biomarkers is increasingly recognized as a better approach to overcome these limitations. For this purpose, genetically manipulated rodent models have been developed to elucidate the etiology and pathogenesis of EOC. However, the artificial nature of the induced tumors in mice limits their clinical relevance [6], [7], [8]. Meanwhile, the laying hen is well known as the only animal that spontaneously develops tumors from ovarian surface epithelium, and the laying hen is a unique and suitable model to develop novel biomarkers and anti-cancer drugs for patients with EOC [6], [7], [9].

*Serpin peptidase inhibitor, clade B, member 3* (*SERPINB3*) is a member of the serpin superfamily of protease inhibitors involved in apoptosis, immune response, blood coagulation, cell migration and invasiveness of cells [10], [11]. It is also known as squamous cell carcinoma antigen 1 (SCCA1) first discovered in squamous cell carcinoma of the cervix [12]. In humans, the gene for *SERPINB3* is located on chromosome 18q21.3, and it inhibits papaine-like lysosomal cysteine proteases, cathepsin K (CTSK), CTSL and CTSS [10], [13]. Previously, we identified *SERPINB3* in chickens and determined that it has moderate homology to its mammalian protein orthologue (approximately 36–47%). Avian SERPINB3 is expressed in the oviduct in response to estrogen in a tissue- and cell-specific manner [14]. Nevertheless, little is known about the expression and prognostic value of *SERPINB3* in either chickens or humans with EOC.

MicroRNAs (miRNAs) are small and non-coding RNAs of 18–23 nucleotides in length. Those regulate gene expression post-transcriptionally and are also able to alter cell fate by controlling translation of target mRNAs in diverse tissues and cell types. Therefore, miRNAs play crucial roles in a various biological processes including vertebrate growth, development, differentiation and oncogenesis by regulating gene expression [15], [16], [17]. However, there are not published results of miRNA research related with SERPINB3 in chickens. In order to determine the role of SERPINB3 as a novel biomarker for EOC, we compared the distribution and localization of SERPINB3 between normal and cancerous ovaries of laying hens, and then we performed a miRNA target validation assay to investigate post-transcriptional regulation of *SERPINB3* expression. After that we compared SERPINB3 expression among normal and cancer cells of ovaries from laying hens, human EOC cell lines (OVCAR-3 and SKOV-3) and a human ovarian teratocarcinoma cell line (PA-1). Finally, we investigated the diagnostic and prognostic values of SERPINB3 expression in patients with EOC.

## Results

### Expression and Localization of SERPINB3 mRNA and Protein in Normal and Cancerous Ovaries of Laying Hens

**Figure 1:**
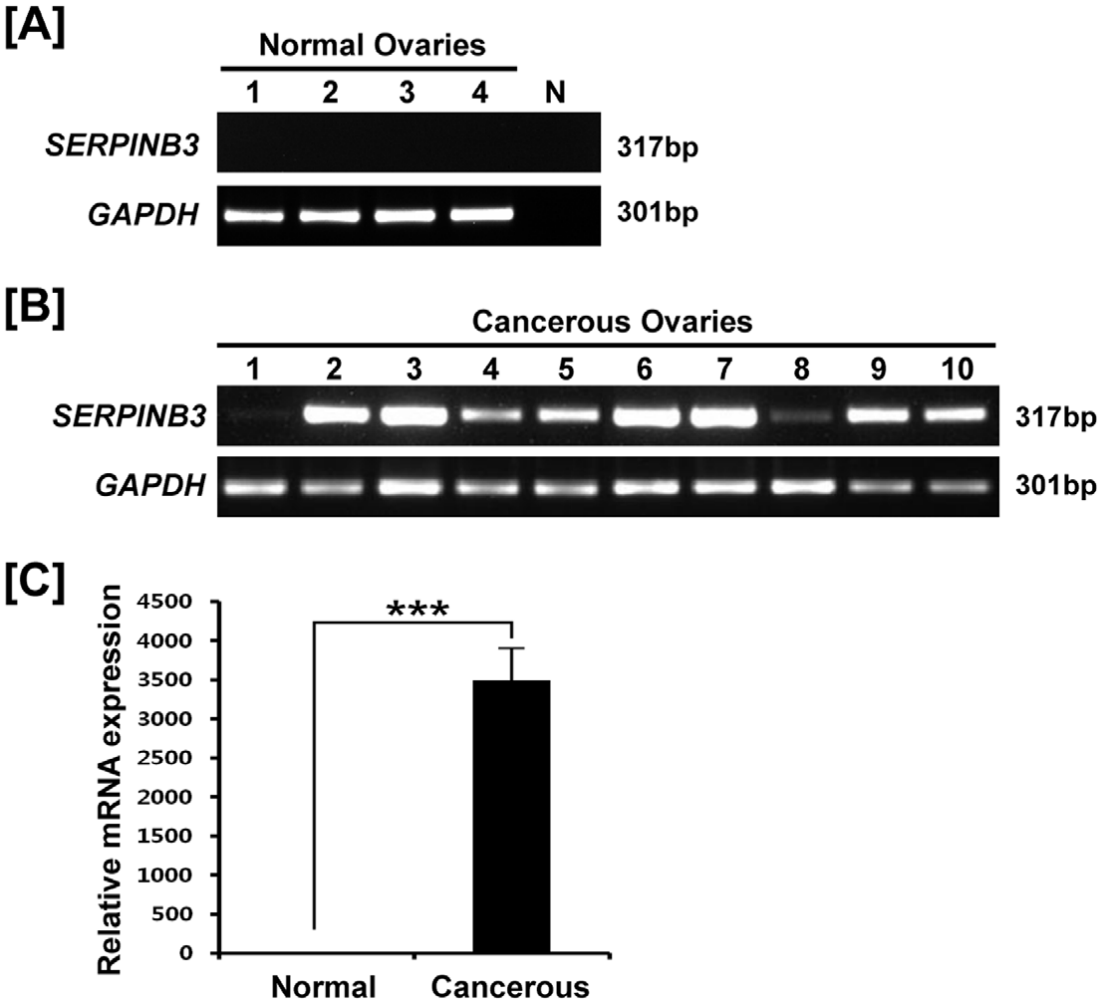
Expression and quantitation of *SERPINB3* mRNA in normal and cancerous ovaries of laying hens. RT-PCR analysis was performed using cDNA templates from chicken *SERPINB3* and *GAPDH*-specific primers. [A] Lanes 1 to 4 show four different normal ovaries and N is negative control. [B] Lanes 1–10 show 10 different cancerous ovaries. [C] Quantitative RT-PCR analysis was performed using cDNA templates from normal and cancerous ovaries of laying hens (mean ± SEM; P<0.01).

Comparisons of expression of *SERPINB3* mRNA between normal and cancerous ovaries of laying hens by RT-PCR revealed that it was expressed in only cancerous ovaries (Fig. 1A and 1B). Moreover, quantitative RT-PCR showed that *SERPINB3* mRNA was induced only in cancerous ovaries (*P*<0.01; Fig. 1C). These results suggest that *SERPINB3* is a biomarker for epithelia-derived ovarian cancer in laying hens.

**Figure 2:**
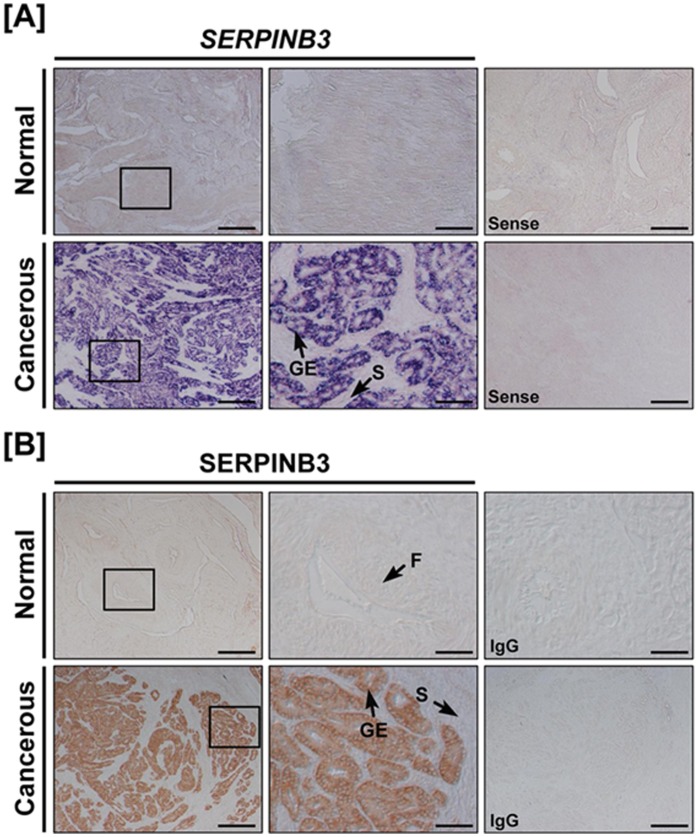
Expression of *SERPINB3* mRNA and protein is unique to glandular epithelium of cancerous ovaries from laying hens. [A] *In situ* hybridization analyses of *SERPINB3* mRNA. Cross-sections of normal and cancerous ovaries from laying hens were hybridized with sense or anti-sense chicken *SERPINB3* cRNA probes. [B] Immunohistochemical expression of SERPINB3 protein: For negative control, the primary antibody was substituted with purified non-immune mouse IgG. F, follicle; GE, glandular epithelium; S, stroma; *Scale bar* represents 200 μm (the first columnar panels and sense) or 50 μm (the second columnar panels).

*In situ* hybridization analysis demonstrated that *SERPINB3* mRNA was abundant in the glandular epithelium of cancerous ovaries of laying hens (Fig. 2A), but was not detectable in the stroma, blood vessels or immune cells of cancerous ovaries. Consistent with these results, SERPINB3 protein was detected predominantly in the cytoplasm of glandular epithelium in cancerous, but not normal ovaries of laying hens (Fig. 2B). These results indicate that SERPINB3 is specifically expressed only in the glandular epithelium of cancerous ovaries of laying hens.

### Post-transcriptional Regulation of microRNAs Affecting SERPINB3

**Figure 3:**
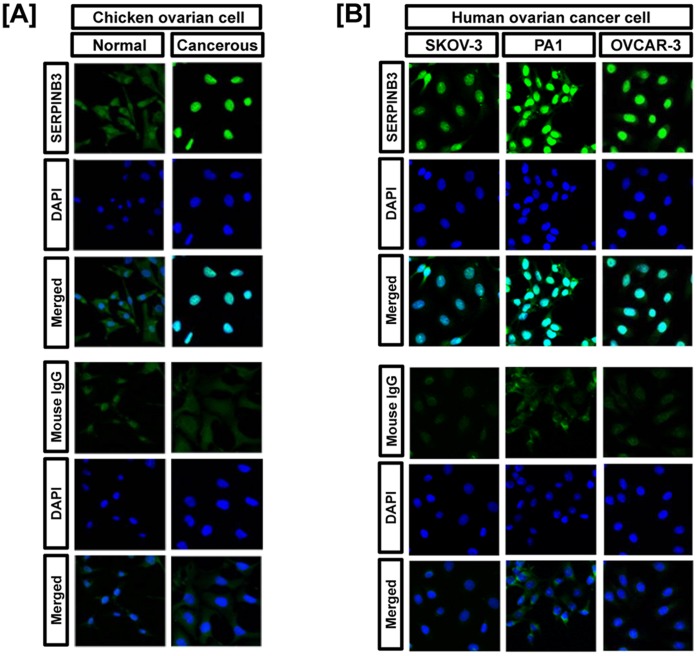
*In vitro* target assay of microRNAs on the SERPINB3 transcript. [A] Diagram of *miR-101, miR-1668* and *miR-1681* binding sites in *SERPINB3* 3′-UTR. [B] Expression vector maps for eGFP with *SERPINB3* 3′-UTR and mutated *SERPINB3* 3′-UTR and Ds-Red with each miRNA. The wild-type (WT) and mutants of 3′-UTR of the *SERPINB3* transcript were subcloned between the eGFP gene and the polyA tail to generate the fusion construct of the GFP transcript following the miRNA target 3′-UTR (pcDNA-eGFP-3′UTR) (top and middle panel) and the miRNA expression vector was designed to co-express DsRed and each miRNA (pcDNA-DsRed-miRNA) (bottom panel). [C] After co-transfection of pcDNA-eGFP-3′UTR for the *SERPINB3* transcript and pcDNA-DsRed-miRNA for the *miR-101, miR-1668* and *miR-1681*, the fluorescence signals of GFP and DsRed were detected using fluorescent microscopy. [D] The rate of inhibition of eGFP expression from miRNA modulation was calculated by fluorescence-activated cell sorting (FACS). Solid bars represent WT of *SERPINB3* 3′-UTR and empty bars show the mutant of *SERPINB3* 3′-UTR for each miRNA. Error bars indicate the standard error of triplicate analyses. The asterisks denote statistically significant differences between WT vs. mutants (***P<0.001).

To investigate the possibility that *SERPINB3* expression is regulated at the post-transcriptional level by miRNAs, we performed a miRNA target validation assay. Analysis of potential miRNA binding sites within the 3′-UTR for *SERPINB3* using a miRNA target prediction database (miRDB; http://mirdb.org/miRDB/) revealed three putative binding site for *miR-101, miR-1668* and *miR-1681* (Figure 3A). Therefore, we determined if these three miRNAs influenced *SERPINB3* expression via its 3′-UTR. A fragment of the *SERPINB3* 3′-UTR harboring binding sites for the miRNAs was cloned downstream of the green fluorescent protein (GFP) reading frame, thereby creating a fluorescent reporter for function of the 3′-UTR region. In addition, SERPINB3 3′-UTR mutants including mutated binding sites for *miR-101*, *miR-1668* and *miR-1681* were also generated by point mutation in order to confirm the modulation of eGFP expression by each miRNA (Figure 3B). After co-transfection of eGFP-*SERPINB3* 3′-UTR and DsRed-miRNA, the intensity of GFP expression and percentage of GFP-expressing cells were analyzed by fluorescence microscopy and FACS (Figure 3C and 3D). In the presence of *miR-101, miR-1668* and *miR-1681*, the intensity and percentage of GFP-expressing cells (44.8% in control vs. 27.5% in *miR-101*, 24.2% in *miR-1668*, 14.8% in *miR-1681*) decreased (p<0.01). However, when there was co-transfection of eGFP-*SERPINB3* 3′-UTR mutants, the intensity and percentage of GFP-expressing cells were not changed (14.8% in control vs. 16.1% in *miR-101*, 13.5% in *miR-1668*, 14.3% in *miR-1681*) as a control (Figure 3D). These results indicate that these three miRNAs directly bind to the *SERPINB3* transcript and post-transcriptionally regulate *SERPINB3* gene expression.

### Immunofluorescence Detection of SERPINB3 Protein in Chicken and Human Ovarian Cancer Cells

**Figure 4:**
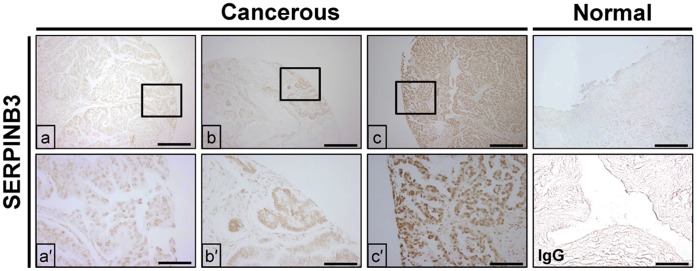
Immunofluorescence microscopy detected SERPINB3 protein in ovarian cancer cell lines of both women and laying hens. [A] SERPINB3 protein was rarely detected in normal cells, but abundant in the nuclei of chicken ovarian cancer cells. [B] SERPINB3 protein was expressed in nuclei of human OVCAR-3, SKOV-3 and PA-1 cells. Cell nuclei were stained with DAPI (blue). All images were captured at 40X objective magnification.

To compare the expression patterns of SERPINB3 protein between chicken and human ovarian cancer cells, we conducted immunofluorescence analysis. SERPINB3 protein was rarely detected in normal cells, but abundant in the nucleus of ovarian cancer cells of laying hens (Fig. 4A). Similarly, SERPINB3 protein was abundant in the nucleus of three human ovarian cancer cell lines, OVCAR-3, SKOV-3 and PA-1 cells (Fig. 4B).

### SERPINB3 Protein Expression is Associated With Platinum Resistance and Survival in Patients with Epithelial Ovarian Cancer

**Figure 5:**
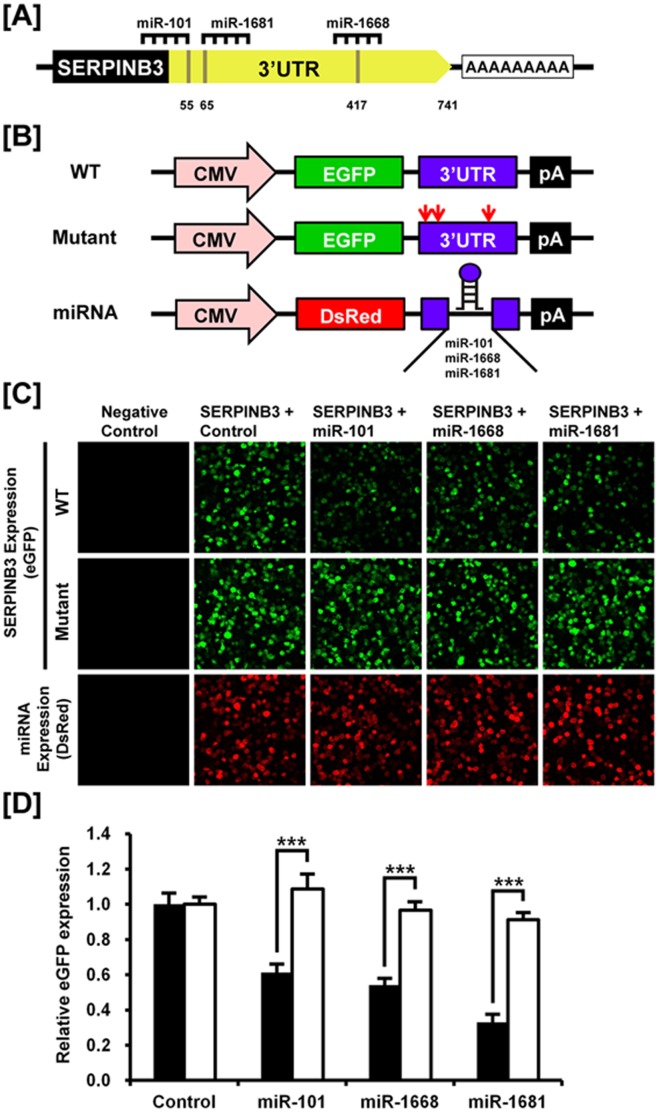
Immunohistochemistry was used to demonstrate expression of SERPINB3 protein in cancerous ovaries, but not in normal ovaries from women. (a and a′) Weak, (b and b′) moderate and (c and c′) strong expression of SERPINB3 protein was detected in women with epithelial ovarian cancer. There was either no expression of very weak expression of SERPINB3 in normal ovaries, whereas SERPINB3 protine was easily detectable in cancerous ovaries from women. The negative control used was mouse IgG instead of primary antibody. *Scale bar* represents 200 μm (the first horizontal panels and IgG) or 50 μm (the second horizontal panels).

A total of 109 patients with a median age of 52 years (range, 23–82 years) were enrolled in the current study. Among all patients, 38 (34.9%) were in FIGO stage I, 20 (18.3%) in stage II, and 51 (46.8%) in stage III. Tumor grade was G1 in 21 (19.3%), G2 in 41 (37.6%) and G3 in 47 patients (43.1%). Histologically, 62 tumors (57%) were diagnosed with serous carcinoma, 17 (15.6%) with mucinous carcinoma, 12 (11%) with clear cell carcinoma, 9 (8.3%) with endometrioid carcinoma, 4 (3.6%) with undifferentiated carcinoma, and 3 (2.7%) with endometrioid and clear cell carcinoma, and 2 (1.8%) with serous and clear cell carcinoma. Optimal cytoreductive surgery was performed in 65 patients (59.6%), whereas 44 (40.4%) underwent suboptimal cytoreductive surgery. Ninety patients (82.6%) received adjuvant chemotherapy after surgery, and 84 (93.3%) received paclitaxel/carboplatin while paclitaxel/cisplatin was administered to 6 (6.7%) patients. Results of immunohistochemistry analysis revealed that SERPINB3 protein was detected predominantly in glandular epithelium as for the laying hen model, and 15 (13.8%), 66 (60.6%) and 28 (25.7%) patients had weak, moderate and strong expression of SERPINB3 protein in ovarian glandular epithelium, respectively (Fig. 5).

The median time to follow-up was 69.2 months (range, 2–88 months), and 70 patients (77.8%) showed platinum sensitivity while 20 (22.2%) demonstrated platinum resistance. Strong expression of SERPINB3 protein was more frequent in patients with platinum resistance (n = 11, 55%) than in those with platinum sensitivity (n = 17, 24.3%) (*P* = 0.01). Strong expression of SERPINB3 protein was also an independent prognostic factor for platinum resistance after adjustments using clinic-pathologic factors (adjusted OR; odds ratio, 5.94; 95% CI, 1.21–29.15) (Table 1). Strong expression of SERPINB3 was associated with shorter PFS than weak or moderate expression (mean PFS were 25.7 vs. 47.8 months; *P* = 0.03) in spite of no difference in OS (mean OS; overall survival, 49.5 vs. 60.9 months; *P*>0.05). Furthermore, SERPINB3 was a poor prognostic factor for PFS (Progression-free survival) using multivariate Cox’s proportional hazard analysis (adjusted HR; hazard ratio, 2.07; 95% CI; confidence interval, 1.03–4.41; Table 2), whereas there was no prognostic factor value for OS except suboptimal cytoreduction (adjusted HR, 6.83; 95% CI, 2.34–20.02).

**Table 1:** Multivariate linear logistic regression analysis for factors affecting platinum resistance.

Characteristics	Adjusted OR^2^	95% CI^3^	p value
Age ≥52 years	0.93	0.25–3.41	0.91
FIGO stage III disease*^1^*	3.06	0.48–19.56	0.24
Grade 3 disease	4.36	1.14–16.71	0.03
Serous adenocarcinoma	0.49	0.11–2.12	0.34
Suboptimal cytoreduction	36.63	5.35–25.07	<0.01
Strong expression of *SERPINB3*	5.94	1.21–29.15	0.03

Abbreviation: FIGO, International Federation of Gynecology and Obstetrics; OR, odd ratio; CI, confidence interval.

**Table 2:** Multivariate Cox’s proportional hazard analysis for poor prognostic factors affecting progression-free survival.

Characteristics	Adjusted HR^2^	95% CI^3^	p value
Age ≥52 years	1.16	0.62–2.17	0.63
FIGO stage III disease^1^	1.49	1.63–3.50	0.01
Serous adenocarcinoma	1.18	0.58–2.40	0.73
Grade 3 disease	1.34	0.76–2.52	0.85
Suboptimal cytoreduction	5.37	2.39–12.07	<0.01
Strong expression of *SERPINB3*	2.07	1.03–4.41	0.04

Abbreviation: FIGO, International Federation of Gynecology and Obstetrics; HR, hazard ratio; CI, confidence interval.

## Discussion

The laying hen is a well-known model for investigation of ovarian carcinogenesis and for development of anti-cancer agents for women because of the similarity in spontaneously arising carcinomas from epithelial ovarian cells [18]. Histology, metastasis and stages of EOC in laying hens are similar to those in humans which indicates the feasibility of using the laying hen model for investigating ovarian carcinogenesis [6]. Furthermore, several biomarkers, including CA-125, are commonly expressed and used for detecting early-stage disease and monitoring therapeutic response in women with EOC and they are cross-reactive with biomarkers for EOC in laying hens [18], [19].

In general, a number of complex glandular architectures are usually found in various carcinomas that arise in various organs such as stomach, bronchus, gladder, prostate, testis and ovary due to the ubiquitous nature of glands. Especially, in ovaries of both avian and mammalian species, these glandular structures are mainly in endometrioid-type tumors with several characteristics such as nuclear atypia, cribriform foci and atresia of stromal follicles. In addition, the glands of adenocarcinomas in women consist of a single layer of epithelial cells undergoing mitosis and sharp luminal margins [6]. Our preliminary results also showed that the glandular architecture in primary ovarian epithelial carcinomas of laying hens is composed of a labyrinth of glands or lacelike papillary folding with large plieomorphic nuclei containing mitotic figures as previously reported [6]. The current study to find a novel prognostic biomarker for patients with EOC using the laying hen model identified high expression of *SERPINB3* gene in glandular epithelium of cancerous ovaries compared to that in normal ovaries from hens. The results suggest that SERPINB3 may be associated with ovarian carcinogenesis through the activation of transcription factors or the inhibition of apoptosis. Our results also revealed that *SERPINB3* gene expression is post-transcriptionally regulated by several miRNAs critical to development of the chicken ovarian carcinogenesis. Furthermore, strong expression of SERPINB3 protein was related to platinum resistance and shorter progression-free survival in patients with EOC. These findings support our hypothesis that SERPINB3 plays a role in tumor development and proliferation from ovarian epithelial cells.

Although the functional role of *SERPINB3* gene in EOC biology is not known, results of the present study indicate clearly that *SERPINB3* is associated with glandular morphogenesis affecting ovarian carcinogenesis in both hens and women with EOC. In both women and hens, SERPINB3 is expressed in the glandular epithelium, but there is little or no SERPINB3 expression in other tissues and cells including stroma and blood vessels of the ovary. These findings indicate that SERPINB3 protein may activate transcription factors or inhibit apoptosis leading to development of EOC in laying hens and women. In previous studies, SERPINB3 was found to regulate programmed cell death by different mechanisms in various cancers and its over-expression was characteristic of cancerous cells of epithelial origin. In addition, SERPINB3 attenuates apoptosis mediated by anti-cancer drugs for NK cells and by inhibiting cytochrome c release from the mitochondria [20], [21], [22] Moreover, glandular morphogenesis associated with *SERPINB3* may contribute to epithelial-mesenchymal transition, which may deregulate the adhesion process to allow metastasis and increase the invasiveness potential of tumor cells in women [23].

Strong expression of SERPINB3 was also associated with platinum resistance and shorter PFS in patients with EOC. The mechanism responsible for development of chemoresistance and a potential role for SERPINB3 has not been established in EOC. Nevertheless, dysfunctional permeabilization of lysosomes contributes to the development of chemoresistance in ovarian cancer cells [24], and SERPINB3 confers resistance to drug-induced apoptosis by inhibiting lysosomal cathepsin proteases in cancer cells [25]. Thus, SERPINB3 may contribute to platinum resistance following chemotherapy-induced lysosomal destabilization. Although strong expression of SERPINB3 was associated with shorter PFS, it is possible that platinum resistance associated with SERPINB3 leads to shorter PFS. This fact is supported by a previous study in which SERPINB3 expression was found to be associated with poor survival in patients with breast cancer [26].

SERPINB3 or SCCA1 has been investigated in various types of squamous cell carcinoma [27], [28], [29], [30], but it has only been suggested as a prognostic factor for breast cancer and lung adenocarcinoma [26], [31]. To our knowledge, results of the current study are significant in being the first to establish the likelihood of a functional role for SERPINB3 in EOC of laying hens. These results validate the laying hen as a model for research on human EOC. Further, the results strongly suggest that SERPINB3 has important functions in development of EOC in laying hens and that it is a novel biomarker for predicting platinum resistance and poor PFS in patients with EOC. Our hypothesis that SERPINB3 has a specific role in development of human EOC requires further evaluation in clinically prospective studies.

## Materials and Methods

### Experimental Animals and Animal Care and Use

The experimental use of chickens in the present study was approved by the Institute of Laboratory Animal Resources, Seoul National University. White Leghorn (WL) laying hens were managed according to approved standards for operation of the University Animal Farm, Seoul National University, Korea. All hens had free *ad libitum* access to feed and water.

### Tissue Samples in Chicken Model

A total 136 laying hens (88 over 36 months and 48 over 24 months of age), which had stopped laying eggs, were euthanized for biopsy and collection of cancerous (n = 10) ovaries. As a control, normal (n = 5) ovaries were collected from egg-laying hens of similar age. We examined tumor stages in 10 hens with cancerous ovaries based on characteristic features of chicken ovarian cancer [6]. Three hens had Stage III EOC as ovarian tumor cells had metastasized to the gastrointestinal tract and liver surface with profuse ascites in the abdominal cavity. In five hens, their tumors had metastasized to distant organs such as liver parenchyma, lung, gastrointestinal tract and oviduct with profuse ascites which is indicative of Stage IV EOC. The other two hens did not have tumors in any other organs; therefore, their ovarian tumors were classified as stage I EOC. Subsets of these samples were fixed in 4% paraformaldehyde for further analyses. After 24 h, fixed tissues were changed to 70% ethanol for 24 h and then dehydrated and embedded in Paraplast-Plus (Leica Microsystems, Wetzlar, Germany). Hens with EOC were classified based on their cellular subtypes and patterns of cellular differentiation with reference to human ovarian malignant tumor types [6].

### RNA Isolation

Total cellular RNA was isolated from frozen tissues using Trizol reagent (Invitrogen, Carlsbad, CA) according to the manufacturer’s recommendations. The quantity and quality of total RNA was determined by spectrometry and denaturing agarose gel electrophoresis, respectively.

### Semi-quantitative RT-PCR Analysis

The expression of *SERPINB3* mRNA in normal and cancerous ovaries of hens was assessed using semi-quantitative RT-PCR as described previously [32]. Complementary DNA (cDNA) was synthesized from total cellular RNA (2 ug) using random hexamer (Invitrogen, Carlsbad, CA) and oligo (dT) primers and AccuPower® RT PreMix (Bioneer, Daejeon, Korea). The cDNA was diluted (1:10) in sterile water before use in PCR. After PCR, equal amounts of reaction product were analyzed using a 1% agarose gel, and PCR products were visualized using ethidium bromide staining.

### Quantitative RT-PCR Analysis

Gene expression levels were measured using SYBR® Green (Sigma, St. Louis, MO, USA) and a StepOnePlus™ Real-Time PCR System (Applied Biosystems, Foster City, CA, USA).[33] The *GAPDH* gene was simultaneously analyzed as a control and used for normalization to account for variation in loading. Each target gene and *GAPDH* was analyzed in triplicate. ROX dye (Invitrogen) was used as a negative control for the fluorescence measurements. Sequence-specific products were identified by generating a melting curve in which the C_T_ value represented the cycle number at which a fluorescent signal was statistically greater than background, and relative gene expression was quantified using the 2^–ΔΔCT^ method [34]. For the control, the relative quantification of gene expression was normalized to the C_T_ of the control ovary.

### In Situ Hybridization Analysis

For hybridization probes, PCR products were generated and were gel-extracted and then cloned into pGEM-T vector (Promega) as described previously [35]. After verification of the sequences, plasmids containing the correct gene sequences were amplified with T7- and SP6-specific primers then digoxigenin (DIG)-labeled RNA probes were transcribed using a DIG RNA labeling kit (Roche Applied Science, Indianapolis, IN). After hybridization and blocking, the sections were incubated overnight with sheep anti-DIG antibody conjugated to alkaline phosphatase (Roche). The signal was visualized by exposure to a solution containing 0.4 mM 5-bromo-4-chloro-3-indolyl phosphate, 0.4 mM nitroblue tetrazolium, and 2 mM levamisole (Sigma).

### MicroRNA Target Validation Assay

The 3′-UTR of *SERPINB3* was cloned and confirmed by sequencing. The 3′-UTR was subcloned between the eGFP gene and the bovine growth hormone (bGH) poly-A tail in pcDNA3eGFP (Clontech, Mountain View, CA) to generate the eGFP-miRNA target 3′-UTR (pcDNA-eGFP-3′-UTR) fusion constructs. In addition, mutants of SERPINB3 3′-UTR for each miRNA were generated by point mutation and then cloned into the same type of plasmids. For the dual fluorescence reporter assay, the fusion constructs containing the DsRed gene and either *miR-101*, *miR-1668* and *miR-1681* were designed to be co-expressed under control of the CMV promoter (pcDNA-DsRed-miRNA). The pcDNA-eGFP-3′-UTR and pcDNA-DsRed-miRNA (4µg) were co-transfected into 293FT cells using the calcium phosphate method. When the DsRed-miRNA is expressed and binds to the target site of the 3′-UTR downstream of the GFP transcript, green fluorescence intensity decreases due to degradation of the GFP transcript. At 48 h post-transfection, dual fluorescence was detected by fluorescence microscopy and calculated by FACSCalibur flow cytometry (BD Biosciences). For flow cytometry, the cells were fixed in 4% paraformaldehyde and analyzed using FlowJo software (Tree Star Inc., Ashland, OR).

### Cell culture

A total of five cell lines, including three human ovarian epithelial cancer and two chicken primary ovarian cells, were used in this study. Human ovarian cancer cell lines (OVCAR-3, SKOV-3, and PA-1) were obtained from the American Type Culture Collection (ATCC, Manassas, VA, USA) and cultured according to supplier’s directions. Two different chicken ovarian surface epithelial cells (normal and cancerous cells) were isolated and cultured as previously described with some modifications [36], [37].

### Immunofluorescence Microscopy for Detection of SERPINB3 Activation

Ovarian cancer cells and normal ovarian cells obtained from laying hens, and three human ovarian cancer cell lines, OVCAR-3, SKOV-3, and PA-1, were examined for SERPINB3 expression patterns by immunofluorescence microscopy as described previously. Each type of cell was seeded onto Lab-Tek chamber slides (Nalge Nunc International, Rochester, NY). After 24 h, cells were fixed with −20°C methanol and immunofluorescence staining was performed using an anti-human SERPINB3 monoclonal antibody (catalog number: ab55733; Abcam plc, Cambridge, UK). Cells were then incubated with Alexa Fluor 488 Rabbit anti-goat IgG secondary antibody (A21222, Invitrogen). Slides were overlayed with DAPI before images were captured using a Zeiss confocal microscope LSM710 (Carl Zeiss) fitted with a digital microscope camera AxioCam and Zen 2009 software.

### Human Study Population

Clinical data were retrieved from a database of patients with EOC between June 2003 and March 2009. Approval by the Institutional Review Board of Seoul National University Bundang Hospital was obtained in advance for the current study. The eligibility criteria were as follows for the patients: they were diagnosed with EOC; they were treated with maximal cytoreductive surgery followed by adjuvant taxane- and platinum-based chemotherapy if indicated; they had Eastern Cooperative Oncology Group performance status of 0–2; and they had no underlying disease affecting survival. _ENREF_4Optimal cytoreductive surgery was defined as a residual tumor ≤1 cm, whereas suboptimal cytoreductive surgery was defined as a residual tumor >1 cm in maximal diameter. All patients except those with low-risk early-stage disease such as FIGO stage IA or IB with grade 1 or 2 disease received adjuvant chemotherapy using paclitaxel (175 mg/m^2^)/carboplatin (AUC 5.0) or paclitaxel (175 mg/m^2^)/cisplatin (75 mg/m^2^) for 1–2 weeks after surgery, and the chemotherapy was repeated every 3 weeks for 6 cycles. Progression-free survival (PFS) was defined as the time elapsed from the date of completion of primary adjuvant chemotherapy to the date of clinically proven aggravation. Overall survival (OS) was calculated from the date of staging laparotomy to the date of cancer-related death or the end of the study. Platinum resistance was defined as the response to platinum-based chemotherapy with a minimum treatment-free interval of less than 6 months while platinum sensitivity was defined as the response to platinum-based chemotherapy with a minimum treatment-free interval of greater than or equal to 6 months.

### Immunohistochemistry

The localization of SERPINB3 protein in normal and cancerous ovaries of hens was evaluated by immunohistochemistry (IHC) _ENREF_21using an anti-human SERPINB3 monoclonal antibody at a final dilution of 1:500 (1 µg/ml), and antigen retrieval was performed using the boiling citrate method as described previously [33]. Negative controls included the substitution of the primary antibody with purified non-immune mouse IgG at the same final concentration. For IHC of human ovarian cancer tissues, representative core tissue sections (2 mm in diameter) were taken from paraffin blocks and arranged in new tissue microarray (TMA) blocks using trephine apparatus (Superbiochips Laboratories, Seoul, Korea). In cases with variable histologic features, the most representative area was selected for TMA construction. The IHC staining of human TMA samples was performed using similar methods for the laying hen model. After IHC, the results were assessed semi-quantitatively by one pathologist unaware of clinico-pathologic characteristics. All EOC tissues showed nuclear staining in most tumor cells, whereas no nuclear staining was observed in normal human ovarian tissues and the negative control where the primary antibody was substituted with purified non-immune mouse IgG at the same concentration. Thus, the staining intensity of tumor cells was graded as weak (1+), moderate (2+) or strong (3+).

### Statistical Analysis

In order to investigate the role of SERPINB3 as a novel biomarker of ovarian carcinogenesis in the laying hen model and to predict clinical outcomes in patients with EOC, data were subjected to analysis of variance, Chi-squared and Student’s *t*-tests, Kaplan-Meier method with the log-rank test, logistic regression and Cox’s proportional hazard analyses to determine odds ratio (OR), hazard ratio (HR), and 95% confidence interval (CI). Statistical analyses were performed using Excel (Microsoft, Redmond, WA, USA) and SPSS software (Version 19.0; SPSS Inc., Chicago, IL, USA). A probability value of *P*<0.05 was considered statistically significant.
