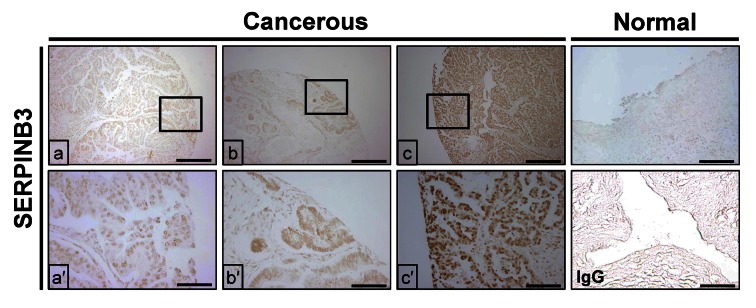# Correction: SERPINB3 in the Chicken Model of Ovarian Cancer: A Prognostic Factor for Platinum Resistance and Survival in Patients with Epithelial Ovarian Cancer

**DOI:** 10.1371/annotation/35f1ffa1-6f3f-42d7-8dc8-e8db569055ed

**Published:** 2013-05-29

**Authors:** Whasun Lim, Hee Seung Kim, Wooyoung Jeong, Suzie E. Ahn, Jinyoung Kim, Yong Beom Kim, Min A. Kim, Mi-Kyung Kim, Hyun Hoon Chung, Yong Sang Song, Fuller W. Bazer, Jae Yong Han, Gwonhwa Song

Figure 3, 4, and 5 were place incorrectly in an article. The legends were all placed correctly.

The correct figures are:

Figure 3: 

**Figure:**
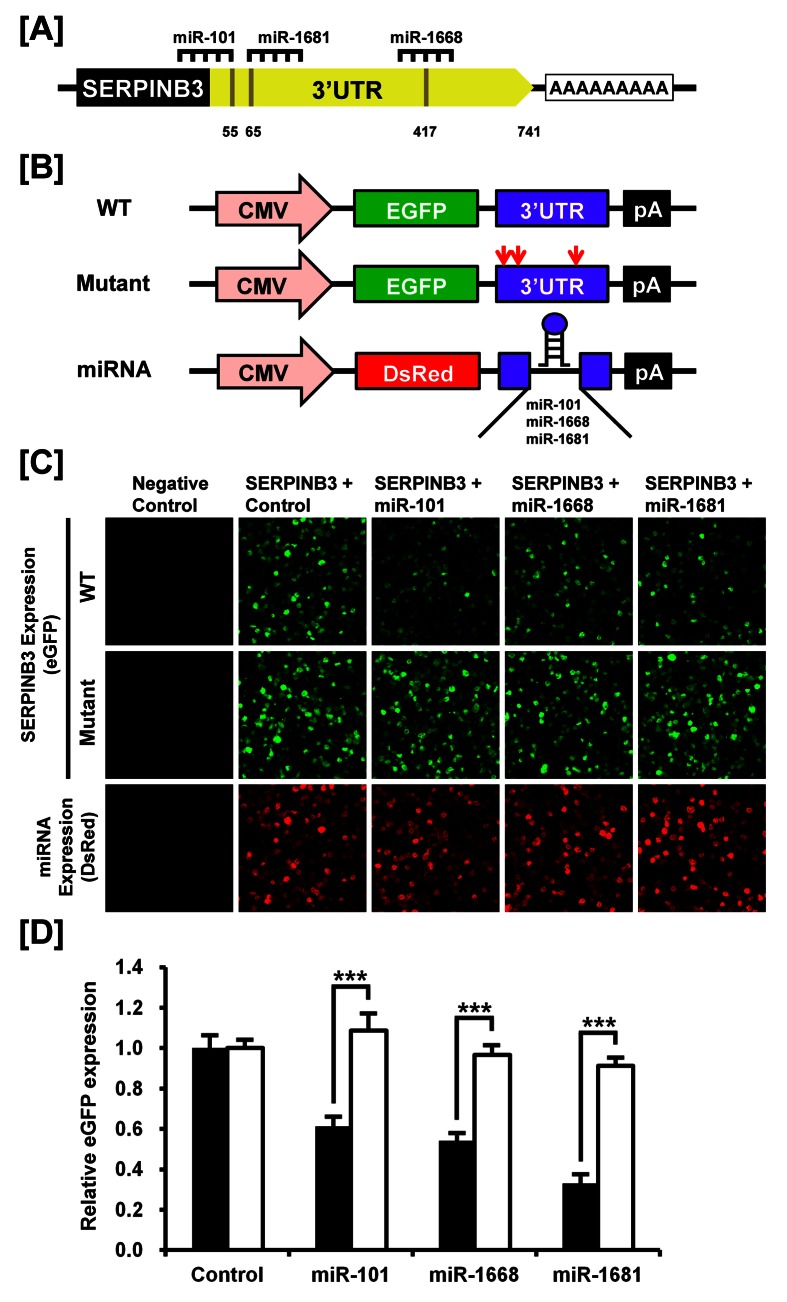


Figure 4: 

**Figure:**
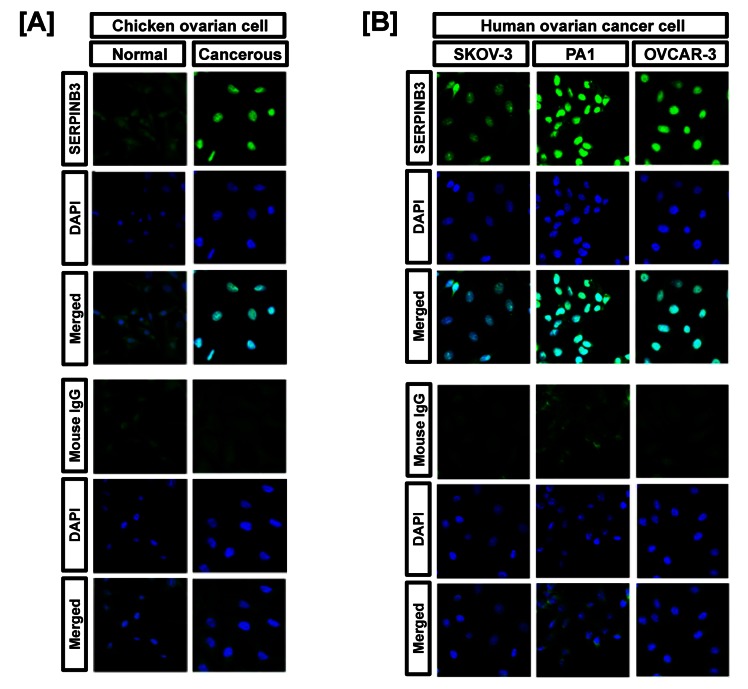


Figure 5: 

**Figure:**